# Rational design of the gram-scale synthesis of nearly monodisperse semiconductor nanocrystals

**DOI:** 10.1186/1556-276X-6-472

**Published:** 2011-07-26

**Authors:** Myriam Protière, Nicolas Nerambourg, Olivier Renard, Peter Reiss

**Affiliations:** 1 DSM/INAC/SPrAM (UMR 5819 CEA-CNRS-UJF)/LEMOH, CEA-Grenoble - 17 rue des Martyrs - 38054 Grenoble cedex 9, France; 2 DRT/LITEN/DTNM/LCSN, CEA-Grenoble - 17 rue des Martyrs - 38054 Grenoble cedex 9 France

**Keywords:** semiconductor nanocrystals, quantum dots, synthesis, experimental plan, fluorescence, scale-up, colloids

## Abstract

We address two aspects of general interest for the chemical synthesis of colloidal semiconductor nanocrystals: (1) the rational design of the synthesis protocol aiming at the optimization of the reaction parameters in a minimum number of experiments; (2) the transfer of the procedure to the gram scale, while maintaining a low size distribution and maximizing the reaction yield. Concerning the first point, the design-of-experiment (DOE) method has been applied to the synthesis of colloidal CdSe nanocrystals. We demonstrate that 16 experiments, analyzed by means of a Taguchi L_16 _table, are sufficient to optimize the reaction parameters for controlling the mean size of the nanocrystals in a large range while keeping the size distribution narrow (5-10%). The DOE method strongly reduces the number of experiments necessary for the optimization as compared to trial-and-error approaches. Furthermore, the Taguchi table analysis reveals the degree of influence of each reaction parameter investigated (e.g., the nature and concentration of reagents, the solvent, the reaction temperature) and indicates the interactions between them. On the basis of these results, the synthesis has been scaled up by a factor of 20. Using a 2-L batch reactor combined with a high-throughput peristaltic pump, different-sized samples of CdSe nanocrystals with yields of 2-3 g per synthesis have been produced without sacrificing the narrow size distribution. In a similar setup, the gram-scale synthesis of CdSe/CdS/ZnS core/shell/shell nanocrystals exhibiting a fluorescence quantum yield of 81% and excellent resistance of the photoluminescence in presence of a fluorescent quencher (aromatic thiol) has been achieved.

**PACS: **81.20.Ka, 81.07.Bc, 78.67.Bf

## Background

The synthesis of colloidal semiconductor nanocrystals has encountered a significant evolution since the seminal work of Murray et al. [1]. In the case of cadmium chalcogenide nanocrystals, for example, the pyrophoric organometallic precursor dimethylcadmium has been replaced by air-stable cadmium oxide [2] and trioctylphosphine oxide (TOPO) has been substituted by a non-coordinating solvent, 1-octadecene (ODE), in combination with a controlled amount of phosphonic or fatty acids acting as stabilizing ligands [3]. These developments are of technological interest for the scale-up of the process. Among the advantages are: the lower price of the starting compounds, their less hazardous and more environmental benign character, and consequently, their easier handling can be listed. However, the comparable development of robust and scalable synthesis methods for size- and shape-controlled nanocrystals of other materials than cadmium chalcogenides still remains a highly challenging task. Generally, the hot-injection method results in the desired separation of nucleation and growth, which is a prerequisite for a low-size distribution [4]. In this synthetic scheme, the mean size of the nanocrystals is governed by the reaction time. On the other hand, stopping the reaction at the desired nanocrystal size before consumption of the precursors leads to low reaction yields and large amounts of waste. Therefore, in order to simultaneously optimize the reaction yield and minimize the size distribution for a given mean size, generally a large set of non-independent reaction parameters (e.g., the nature and concentration of precursors and stabilizing ligands, the solvent composition, or the reaction temperature) has to be carefully adjusted. Although this problem is generic for the development of any new synthesis, no systematic method can be found in literature. A straightforward trial-and-error approach has a high risk of being time and chemical consuming due to the large number of parameters to be optimized. The design-of-experiment (DOE) method is an efficient generic way for the rapid optimization of synthesis methods [5,6]. Its systematic approach leads to a considerably reduced number of experiments as compared to the intuitive trial-and-error method. While routinely used in chemical engineering, the application of this procedure to the rational design of semiconductor nanocrystals' synthesis has not been reported to date. Literature examples include its use in organic synthesis [7,8], in the catalytic chemical vapor deposition (CCVD) growth of single-wall carbon nanotubes [9], or in the synthesis of nano-sized TiO_2 _[10], and silver particles [11].

In this contribution, we evaluate the potential of the DOE approach using the Taguchi method of experimental plans for the optimization of the synthesis of semiconductor nanocrystals [5]. We chose CdSe nanocrystals as a model system, taking advantage of the reported correlations between their size/size distribution and their optical properties [12,13]. The DOE method allowed us with a minimum number of experiments to deduce the degree of influence of each individual reaction parameter on the nanocrystals' mean size and size distribution and indicated interactions between the parameters. We present the results of the matrix cross-analysis of 16 experiments, which served as the basis for the subsequent determination of optimum synthesis parameters of three different-sized, nearly monodisperse samples of CdSe nanocrystals. Our study reveals that the DOE method is a useful tool for the rapid optimization of nanocrystal synthesis methods. On the other hand, we demonstrate its limits in terms of predictivity, which strongly depends on the initially defined experimental domain and on the interactions between experimental parameters. The developed synthetic protocol can easily be scaled up without sacrificing the narrow size distribution of the samples. This is shown in the second part of the article, where we describe the experimental setup and conditions for the gram-scale synthesis of CdSe core and of highly luminescent CdSe/CdS/ZnS core/shell/shell nanocrystals.

## Results and discussion

### Building up the experimental plan

One of the basic characteristics of the DOE technique is the fact that the level of each factor (i.e., reaction parameter) is varied in each experiment according to a *predefined plan*. Its application can be divided into the following, subsequent steps:

1. Choice of the factors to be investigated and varied;

2. Definition of the experimental domain, i.e., the range of values each factor can have;

3. Choice of the interactions between the factors to be investigated;

4. Choice of the table used for the analysis.

In order to evaluate the potential of the DOE method for the optimization of a synthesis protocol for semiconductor nanocrystals, we choose the procedure proposed by Yu and Peng [3] for the preparation of CdSe nanocrystals in ODE. This method has a high potential for the large-scale production of nanocrystals, as inexpensive and easily to manipulate precursors and solvents are used. The objective of our optimization was to identify the reaction parameters for the synthesis of nearly monodisperse (σ = 5-10%) samples of a given mean size while simultaneously maximizing the reaction yield. The latter condition implies that the reaction is only stopped after nanocrystals' size evolution has reached a plateau with negligible further change of the mean size for longer reaction times. Stopping at an earlier stage unavoidably reduces the yield as a significant amount of unreacted precursors remain in the reaction mixture. The size and size dispersion of the CdSe nanocrystals were determined using the correlation between their diameter and position of the excitonic peak in the UV-vis absorption spectrum [12], and the empiric relationship between the size distribution and the photoluminescence (PL) linewidth (full width at half maximum (FWHM)) established in Ref. [13], respectively. In this context, it has to be noted that the chosen reaction system using fatty acid-type stabilizing ligands instead of phosphonic acids [2] generally leads to nearly perfectly spherical particles, which reduces a possible impact of shape dispersion on the PL linewidth. Therefore, variations in the crystal diameter can be considered as the only source of line broadening. For a number of samples, the mean size and size distribution have additionally been assessed by means of transmission electron microscopy (TEM). Table 1 lists the factors chosen for investigation within the experimental plan as well as the levels defined for each factor. In the case of a completely new system lacking previous knowledge, the choice of the factors and their levels would require the conduction of some preliminary experiments prior to establishing the experimental plan.

**Table 1:** Definition of the factors A-G and levels of each factor

		Level 1	Level 2
A	Solvent	ODE	ODE/oleylamine
B	[Cd]	10 mmol/l	50 mmol/l
C	T	250°C	300°C
D	Se/Cd	1:2	5:1
E	Ligand/Cd	8:1	25:1
F	Ligand	Stearic acid (SA)	Oleic acid (OA)
G	Se precursor	TBP-Se	TOP-Se

ODE: 1-octadecene; TBP: tributylphosphine; TOP: trioctylphosphine.

With the goal to minimize the number of experiments, we limited our study to only two levels per factor. The latter should be defined as "extreme" values in order to get information about the maximum obtainable nanocrystals' size range with the chosen synthesis method and to enhance the influence of possible interactions between the factors. At the same time, the experimental domain has to be kept narrow enough to enable a linear extrapolation to expected results for values used in between the predefined levels.

While the used levels of concentration, temperature and precursor ratio are based on literature examples describing this system [3,14], the choice of the other levels has to be explained in more detail. Concerning the solvent, the use of a primary amine such as oleylamine as an additive to pure ODE has been motivated by the fact that these compounds have been shown to narrow the size distribution of CdSe nanocrystals [15,16]. Furthermore, an efficient passivation of surface trap states by primary amines has been observed, leading to increased PL efficiencies [15,17-19]. In terms of stabilizing ligands, both oleic acid and stearic acid have been reported in ODE solvent. These molecules only differ in the double bond within the alkyl chain of the former. However, it cannot be excluded that this double bond is implied in the reaction mechanism, as for example observed in the case of FePt nanoparticles [20]. Concerning the selenium precursor, elemental selenium dissolved in either trioctylphosphine (TOP) or tributylphosphine (TBP) are most widely used in the literature. An influence of the length of the phosphine alkyl chains on the reaction kinetics and hence on the nanocrystals' mean size can be expected due to the different steric hindrance of both molecules.

If it was attempted to analyze the effect of each factor and interaction within a complete experimental plan, 2^7 ^= 128 experiments had to be carried out (seven factors at two different levels). This number could be significantly reduced by studying only the most relevant interactions between the defined factors, which - in view of the current state of knowledge - have been considered to be BF, DE, DF, DG, EF, EG, FG [3,15,17,21-23]. Taguchi tables are predefined tables used to identify the individual experiments according to the number of factors and interactions to be studied and the desired resolution [5]. Here, the fractional Taguchi table L_16 _was chosen, which allows for the determination of the effect of each factor and of the selected interactions with only 16 experiments, as shown in Table 2. This plan determines the level of each factor for each experiment (cf. Table 1). It should be noted that the effect of each factor is obtained without aliasing, whereas the second order interactions are connected two by two.

**Table 2:** Taguchi table L_16 _used for the optimization of seven individual experimental parameters

Trial	A	B	C	D	E	F	G
1	1	1	1	1	1	1	1
2	1	1	1	1	2	2	2
3	1	1	2	2	1	1	2
4	1	1	2	2	2	2	1
5	1	2	1	2	1	2	2
6	1	2	1	2	2	1	1
7	1	2	2	1	1	2	1
8	1	2	2	1	2	1	2
9	2	1	1	2	1	2	1
10	2	1	1	2	2	1	2
11	2	1	2	1	1	2	2
12	2	1	2	1	2	1	1
13	2	2	1	1	1	1	2
14	2	2	1	1	2	2	1
15	2	2	2	2	1	1	1
16	2	2	2	2	2	2	2

The factors A-G and their levels 1/2 are defined in Table 1.

### Analysis of the experimental plan

The results of all 16 experiments, carried out following the conditions defined in Tables 1 and 2, are summarized in Table 3.

**Table 3:** Properties of the nanocrystals obtained with the experimental parameters defined in Tables 1 and 2

Trial	λ_abs _(nm)	Size (nm)	λ_PL _(nm)	FWHM (nm)
1	610	5.1	628	36
2	596	4.4	611	34.2
3	618	5.5	620	44.9
4	618	5.5	635	48.8
5	590	4.2	584	55.6
6	638	6.8	642	40
7	620	5.6	632	37.9
8	618	5.5	625	30.1
9	584	4.0	593	62.2
10	612	5.2	614	46.3
11	582	3.9	599	43.3
12	618	5.5	621	34.2
13	596	4.4	637	61
14	630	6.2	652	33.9
15	600	5.3	613	43
16	596	4.4	598	62.8

The fact that each experiment led to the formation of CdSe nanocrystals is a first indication of the correct choice of the explored experimental domain. Different calculations have to be made in order to analyze the experimental plan [5].

(a) Calculation of the *average effect *of each factor at each level with the relation:(1)

*E_Ai_*: effect of factor *A *in level i

*y_Ai_*: response when factor *A *is in level i

*y*_0_: average of the responses of all experiments

We see that the effect of the factor *X *at the level 1 is the opposite of the effect of the factor *X *at the level 2.

(b) Calculation of the *predefined interactions*:(2)

*I_AiBj _*is the interaction of factor *A *in level i and factor *B *in level j.

*y_AiBj _*is the response when factor *A *is in level i and factor *B *in level j.

*y*_0 _is the average of the responses of all experiments.

(c) Building of the models

As mentioned before, the experimental results were primarily analyzed in terms of the obtained size distribution (correlated with the PL FWHM) and of the mean size. Having calculated the effects and interactions with Eqs. 2 and 3 and using the values obtained experimentally (cf. Table 3), models for the *prediction *of the mean size and size distribution can be built.

These models are of the general form:(3)

Equation 3 results in the following model for the PL FWHM:

In the order of significance, the strongest influence on the emission linewidth was observed for the factors D (Se/Cd ratio), A (solvent), and E (ligand/Cd ratio). The influences of all factors are summarized in Table 4. A Se/Cd ratio of 1:2 leads to more monodisperse nanocrystals than a value of 5:1. This behavior is in discrepancy with the results reported for the synthesis of CdSe nanocrystals in the coordinating solvent TOPO with an admixture of hexadecylamine [22]. In the case of the non-coordinating solvent ODE, the cadmium precursor exhibits a higher reactivity and therefore a Se-limited reaction leads to a better control of the nanocrystals' growth rate. Similarly, the S/Cd ratio yielding monodisperse CdS nanocrystals in ODE has been reported to be within the range of 1:1.5 and 1:10 [3,24-26], whereas in coordinating solvent it lies between 4:1 [27] and 1:1.3 [1]. The ratio [stabilizing ligand]:[Cd] has been identified being a crucial factor for the control of the size dispersion [3]. It directly influences the balance between nucleation and growth by changing the reactivity of the Cd precursor. In particular, the increase of the ligand/Cd ratio leads to a slower growth rate due to the increased steric hindrance of the Cd precursor and thus to a reduced size distribution. Introducing oleylamine into the system results in an increase of the photoluminescence intensity, which is however accompanied by a broadening of the linewidth.

**Table 4:** Influence of each factor on nanocrystals' PL linewidth (FWHM) without considering interactions between parameters

		Level 1	Level 2
A	Solvent	-	+
B	[Cd]	-	+
C	T	+	-
D	Se/Cd	-	+
E	Ligand/Cd	+	-
F	Ligand	-	+
G	Se precursor	-	+

"+" means that increasing the value of this factor leads to an increased linewidth.

The analysis of the experimental plan further indicates that the only important interaction between experimental parameters is that between the Se/Cd ratio and the type of the ligand (DF). This dependence is not unexpected because the reactivity of the cadmium precursor complex depends on the strength of the Cd-ligand coordination bond. Here, the observed difference in reactivity between the Cd-stearate and the Cd-oleate complex points at the implication of the double bond in the carbonaceous chain of oleic acid during the complex formation.

Equation 3 gives the following model for the nanocrystals (NC) mean size:

The nanocrystals' size is mainly affected, in the order of significance, by the type of the Se precursor (G), the ligand/Cd ratio (E), and the type of the ligand (F) (cf. Table 5). The use of TBP-Se as the Se source leads to larger nanocrystals than the use of TOP-Se. This behavior seems to be counter-intuitive at a first glance, when considering that the decrease of the alkyl chain length in strongly *Cd-coordinating *ligands, such as phosphonic acids, leads to smaller nanocrystals [28]. In the case of *Se-coordinating *phosphines, the lower reactivity of TBP-Se as compared to TOP-Se most probably counterbalances steric effects, leading to the formation of fewer seeds during the nucleation phase, which finally results in a larger particle size [29]. As expected, the ligand/Cd ratio strongly affects the growth kinetics of the nanocrystals. In non-coordinating solvents, the reactivity of the cationic monomers, i.e., the cadmium complexes which are not yet deposited onto the surface of the growing crystallites, is strongly dependent on the ligand concentration [3]. In accordance with classical crystallization theory, the higher the ligand/Cd ratio, the lower the number of nuclei formed during the nucleation phase due to the decreased monomer reactivity, resulting after the growth stage in nanocrystals of a larger mean size. With the given reactants and experimental domain explored, we did not succeed in synthesizing nanocrystals with a size smaller than 3.7 nm. This is coherent with reported results, indicating that fatty acid-type stabilizers lead to fast growth rates and to comparably large nanocrystals [21]. The presence of a double bond in the carbonaceous chain of the stabilizer results in a strong influence on the nanocrystal size with oleic acid leading to smaller particles than stearic acid. Adding a small amount of oleylamine to the reaction mixture leads to a small size decrease. The results of the DOE analysis reveal that the most relevant interactions between parameters concerning both the size and the emission linewidth are the interactions between the Se/Cd ratio and the type of the stabilizing ligand. Table 6 lists, for all 16 experiments, the experimental values of the size and linewidth and compares them to the values calculated with the models. The error levels of less than 6% for the size and 12% for the linewidth illustrate the good accordance between the experiment and the model and validate the assumptions made. It has to be underlined that this agreement is even observed in trials resulting in comparably polydisperse samples (trials 9, 13, and 16).

**Table 5:** Influence of each factor on the nanocrystal size without considering interactions between the parameters

		Level 1	Level 2
A	Solvent	+	-
B	[Cd]	-	+
C	T	-	+
D	Se/Cd	-	+
E	Ligand/Cd	-	+
F	Ligand	+	-
G	Se precursor	+	-

"+" means that increasing the value of this factor leads to a size increase.

**Table 6:** Comparison of the experimental and calculated values for size and linewidth for the 16 experiments

Trial	Experimental size	Calculated size	Error (%)	Trial	Experimental FWHM	Calculated FWHM	Error (%)
1	5.06	5.08	0.43	1	36	38.31	6.03
2	4.41	4.39	-0.48	2	34.2	31.89	-7.24
3	5.49	5.54	0.9	3	44.9	42.19	-6.42
4	5.49	5.44	-0.92	4	48.8	51.51	5.26
5	4.17	4.36	4.31	5	55.6	56.94	2.35
6	6.80	6.61	-2.84	6	40	38.66	-3.47
7	5.61	5.82	3.68	7	37.9	34.21	-10.79
8	5.49	5.28	-3.98	8	30.1	33.79	10.92
9	3.95	3.76	-5.16	9	62.2	60.86	-2.2
10	5.16	5.35	3.51	10	46.3	47.64	2.81
11	3.89	3.67	-5.89	11	43.3	46.99	7.85
12	5.49	5.7	3.68	12	34.2	30.51	-12.09
13	4.41	4.39	-0.48	13	61	58.69	-3.94
14	6.23	6.26	0.43	14	33.9	36.21	6.38
15	5.27	5.22	-0.92	15	43	45.71	5.93
16	4.41	4.46	1.1	16	62.8	60.09	-4.51

In order to verify that the models are valid within the entire experimental domain, complementary trials have been carried out, in which different combinations of the levels 1 or 2 for each factor were chosen with respect to the initial experimental plan (cf. Table 7). The error levels of experiments 17 and 18 confirm the good reliability of the models, even though the error for the size in trial 18 (16%) is significantly higher than in all other trials.

**Table 7:** Summary table of the complementary trials used to verify the models

Trial	A	B	C	D	E	F	G	Experimental size	Calculated size	% error	Experimental FWHM	Calculated FWHM	% error
17	2	1	1	2	2	2	2	3.31	3.67	9.8	57.7	62.7	8.8
18	1	2	2	1	1	1	2	4.17	4.96	15.9	48.3	48.3	0

### Selection of synthesis parameters for three different-sized samples with narrow size dispersion

The knowledge of the influence of the different experimental parameters on the nanocrystals' size and size distribution was subsequently used for the design of the synthesis of three different-sized samples with a minimum spectral overlap of emission. The experimental parameters used are summarized in Table 8.

**Table 8:** Choice of the experimental parameters for the synthesis of CdSe nanocrystals of three different sizes

Factor	Nanocrystals' size		
	Small (3.6 nm)	Medium (4.5 nm)	Large (6.5 nm)
Solvent	> 2	> 2	1
[Cd]	1	1	> 2
T	1	1	2
Se:Cd	2	2	1
Ligand:Cd	2	2	> 2
Ligand	1	1	1
Se precursor	2	2	2

Experimental parameters for three different sizes while optimizing the reaction yield and minimizing the size dispersion. "> 2" signifies that the initially chosen level of this parameter is not appropriate to obtain the desired response.

**Figure 1:**
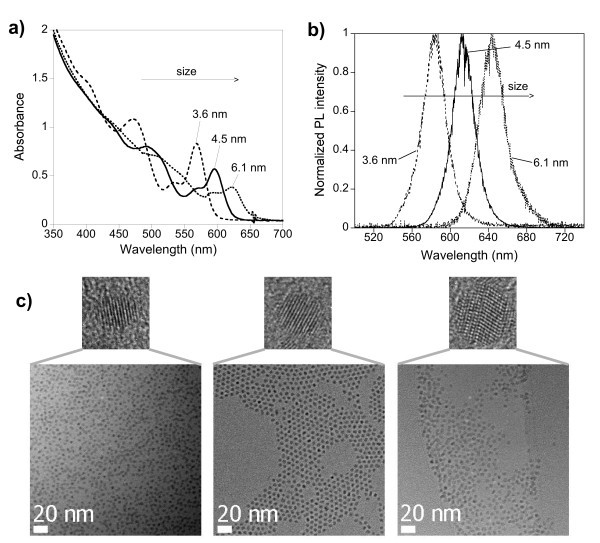
**Absorption, PL spectra, and TEM images of the three samples obtained**. UV-vis absorption (**a**) and normalized photoluminescence (**b**) spectra of the three different-sized samples (excitation wavelength, 400 nm). (**c**) TEM images: 3.6-, 4.5-, and 6.1-nm CdSe nanocrystals (from left to right) at identical magnification and high resolution images of single particles (image size 7 × 7 nm).

The results of the DOE analysis point at the combination A2, B1, C1, D1, E1, F2, and G2 for obtaining the smallest size of nanocrystals. However, stearic acid was preferred over oleic acid with the goal to minimize the synthesis cost (level F = 1). The experiment with the combination A2, B1, C1, D1, E1, F1, and G2 led indeed to small nanocrystals (3.3 nm diameter) but the size dispersion was unacceptable as indicated by the FWHM (43 nm). With the goal to decrease the size dispersion, the parameters D and E were set to level 2, as (1) the interaction DF has a strong influence on the FWHM and (2) increasing the ligand/Cd ratio tends to lower the FWHM (*vide supra*). The resulting combination (A2, B1, C1, D2, E2, F1, and G2) has already been tested (trial 10) and led to rather large nanocrystals (5.2 nm) having an important size dispersion (FWHM 46 nm). These results indicate that the experimental domain for one or several parameters have been chosen too narrow in view of obtaining low size distributions. The molar quantity of oleylamine is a parameter, which had been - in contrast to all other parameters - rather arbitrarily set to 25% of the molar quantity of the stabilizing ligand (stearic or oleic acid), due to the absence of appropriate literature results. Therefore the value fixed in the experimental plan may lie outside the optimum range. Using the same molar quantity of oleylamine and stearic acid (10 mmol) led to nanocrystals with a size of 3.6 nm ("small size") and a FWHM of 28-29 nm. Alkylamines are known to "activate" metal carboxylate complexes in the synthesis of II-VI and III-V nanocrystals [30,31]. Therefore, in order to obtain larger nanocrystals ("medium size"), the quantity of oleylamine has been significantly increased to 42.5 mmol, resulting in particles with a diameter of 4.5 nm and an average FWHM of 27.5 nm. The reaction yields were 95% (3.6 nm) and 80% (4.5 nm), i.e., in both cases the conversion of the minority precursor (Cd) is close to quantitative. The synthesis of even bigger nanocrystals ("large size") is based on the parameters used in experiment 8, which resulted in 5.5-nm particles with a FWHM of 30 nm. Increasing at the same time the cadmium concentration and the ligand:Cd ratio by a factor of 2 led to 6.1-nm nanocrystals (FWHM 30 nm). However, working with a large ligand/Cd ratio of 50:1 in the absence of oleylamine reduced the reactivity and conversion of the cadmium precursor and therefore the reaction yield was only 60% in this case. The absorption and PL spectra as well as TEM images of the three samples obtained are depicted in Figure 1.

Concluding this part, we have shown that the applied experimental plan can be used to identify within a comparably low number of experiments the influence of the different reaction parameters on the nanocrystals' size and size dispersion and to identify the most important interactions between parameters. The values obtained with models built for calculating/predicting the size and PL linewidth of the nanocrystals are in good agreement with the experimental values, validating the assumptions made. Therefore, the synthesis of nanocrystals having a desired mean size and low size dispersion can be designed on the basis of the results of the experimental plan. On the other hand, the predictivity of the experimental plan is strongly dependent on the chosen experimental domain, i.e., the levels of the parameters (factors). As a consequence, extrapolation of the levels of some parameters beyond the predefined experimental range may be necessary. The use of an experimental plan having three (or more) levels per parameter reduces this problem.

### Scale-up of the synthesis

**Figure 2:**
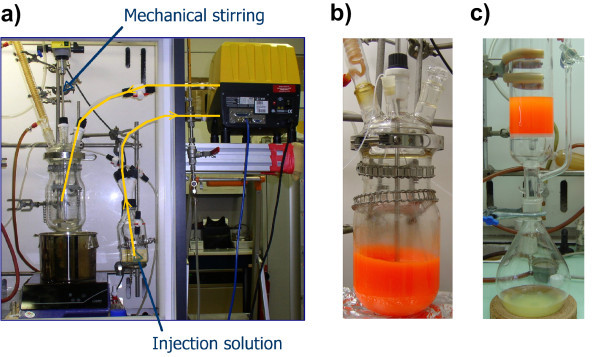
**Experimental setup**. (**a**) Experimental setup used for the gram-scale synthesis of nearly monodisperse CdSe nanocrystals. (**b**) CdSe/CdS/ZnS core/shell/shell nanocrystals in the 2-L reactor at the final stage of synthesis. (**c**) Purification using a glass filter column retaining the precipitated nanocrystals, while the solvent and byproducts are collected in the round-bottom flask below.

A laboratory-scale synthesis as described in the last paragraphs yields approximately 100-200 mg of CdSe nanocrystals. Their potential technological applications make the scale-up of the synthesis highly desirable. Several attempts in this direction have been reported in the recent literature [32-35]. However, the detailed description of the experimental setup and the comparison of the results obtained with the gram scale and with the laboratory-scale synthesis are lacking in all cases. In a straightforward approach, we carried out the three synthesis protocols developed precedently in a 2-L reactor, after multiplying the quantities of all reagents by a factor of 20. Special care was taken concerning the stirring and the injection, both of which being of crucial importance for the size control of the nanocrystals. Figure 2a shows the experimental setup composed of the 2-L reactor, a mechanical stirrer, a 500-mL bottle containing the injection solution (TOPSe, ODE) and a Watson-Marlow 620 DI/RE peristaltic pump (Watson-Marlow and Bredel Products, La Queue Lez Yvelines, France) on the right, capable of injecting up to 500 mL within 3.5 seconds. The cover of the reactor contains five inlets used for the stirring bar, the condenser, the temperature probe, the injection tube, and for taking aliquots. The 500-mL bottle is equipped with a Teflon cap containing four inlets, allowing at the same time for the argon circulation and passage of the injection solution. The whole setup is connected to a vacuum line and can be used under inert atmosphere. While all other steps were carried out in the same way as during the laboratory-scale synthesis, purification was achieved by filtration rather than by centrifugation. It turned out to be impracticable to purify large quantities of nanocrystals by centrifugation due to the concomitant precipitation of large amounts of stearic acid. In order to avoid this phenomenon, the temperature of the reaction mixture is kept above 70°C after addition of acetone/ethanol. For the filtration, a standard glass filter (G3) is used, which allows carrying out the purification under inert atmosphere.

**Figure 3:**
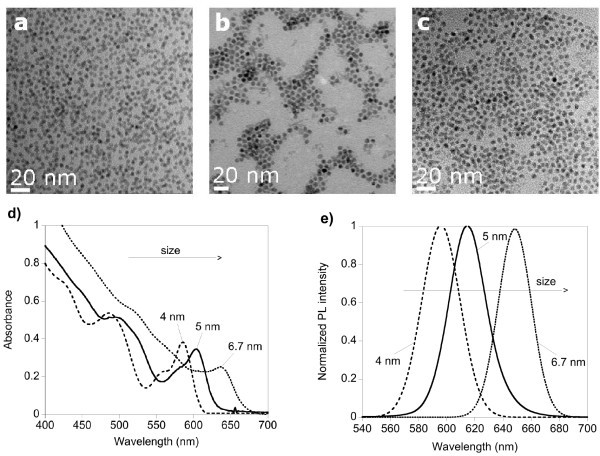
**TEM images and spectra of the final, purified samples**. (**a**, **b**, **c**) TEM images of the obtained 4.0-, 5.0-, and 6.7-nm CdSe nanocrystals (from left to right). (**d**) Corresponding UV-vis absorption spectra; (**e**) normalized PL spectra (excitation wavelength, 450 nm).

The evolution of the absorption and PL spectra of aliquots taken during the gram-scale synthesis is very similar to the laboratory-scale synthesis. The spectra of the final, purified samples (Figure 3d or 3e) exhibit well-defined excitonic peaks in UV-vis absorption and narrow emission linewidths (FWHM 30-32 nm). The narrow size distribution of approximately 7.5% for all three samples is confirmed in the TEM images Figure 3a, b, c. We attribute the general observed size increase in the gram-scale synthesis to the fact that the powerful mechanical stirring influences the nucleation and growth kinetics as compared to the magnetically stirred laboratory-scale synthesis.

### Gram-scale synthesis of CdSe/CdS/ZnS core/shell/shell nanocrystals

**Figure 4:**
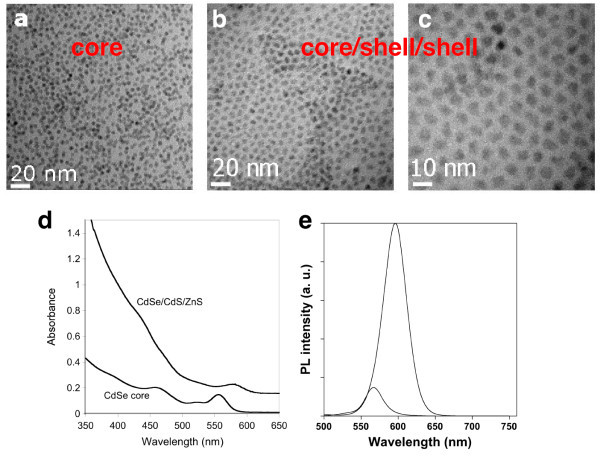
**TEM images, UV-vis absorption spectra, and PL spectra of core and core/shell/shell nanocrystals**. TEM images of 4-nm CdSe core (a) and of the corresponding 6.2-nm CdSe/CdS/ZnS core/shell/shell nanocrystals (b) at the same magnification; (c) at higher magnification. d) UV-vis absorption spectra (the spectrum of the core/shell/shell nanocrystals has been shifted vertically for clarity); e) PL spectra (excitation wavelength, 400 nm) of the core and core/shell/shell nanocrystals.

In order to improve the stability against photobleaching and the quantum yield of the obtained CdSe nanocrystals, their surface was subsequently passivated with a CdS/ZnS double shell. The intermediate CdS shell serves as "lattice mismatch mediator", reducing the strain between the CdSe core and the ZnS outer shell [36,37]. The same reactor was used as in the core nanocrystal synthesis, while a syringe pump replaced the peristaltic pump for the slow injection of the shell precursors. The latter were composed of a mixture of cadmium ethylxanthate and cadmium stearate for the CdS shell and of zinc ethylxanthate/zinc stearate for the ZnS shell, respectively [26]. During the shell growth, a red shift of the absorption (from 556 to 582 nm) and PL (from 573 to 592 nm) peaks is observed, indicating the partial delocalization of the exciton in the shell (Figure 4d or 4e). The PL FWHM increases from 32 to 37 nm during the shell growth, going along with the enlargement of the size dispersion from 7.5% to 10%. The fluorescence quantum yield (QY) of the obtained CdSe/CdS/ZnS nanocrystals accounts for 81%, i.e., shell growth led to an increase of the PL QY by a factor of 10. In contrast to the used rather spherical CdSe core nanocrystals, the core/shell/shell particles have a more facetted shape. On TEM images (Figure 4a, b, c), a size difference of 2.2 nm between the core and core/shell/shell nanocrystals has been determined corresponding to three to four shell monolayers. This size increase is expected in view of the quantity of the injected shell precursors (calculated for obtaining 1.3 CdS monolayers and 2.5 ZnS monolayers), indicating that essentially the whole amount of precursors has been deposited on the core nanocrystals.

**Figure 5:**
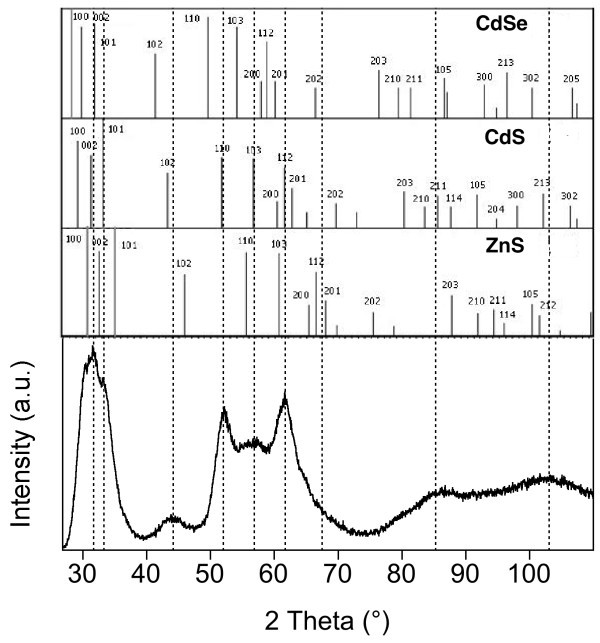
**Powder X-ray diffractogram of the CdSe/CdS/ZnS sample**. The diffraction pattern of the sample (dashed lines) is compared with the peak positions of bulk wurtzite CdSe (JCPDS file: 8-0459), CdS (JCPDS file: 80-0006) and ZnS (JCPDS file: 80-0007).

**Figure 6:**
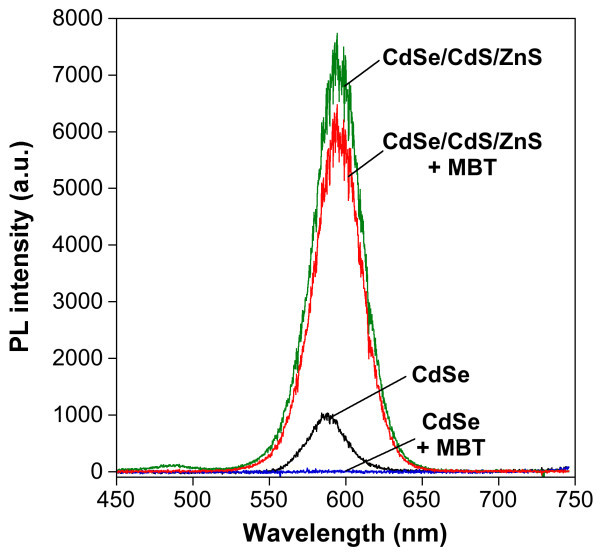
**PL intensity in presence of a fluorescence quencher**. Comparison of PL intensity before and after addition of 0.015 mL of a 0.5 M solution of 4-methylbenzenethiol (MBT) in chloroform to CdSe core and CdSe/CdS/ZnS core/shell/shell nanocrystals in hexanes (1 mL; excitation wavelength, 400 nm).

The powder X-ray diffractagramm of the CdSe/CdS/ZnS nanocrystals (Figure 5) exhibits peaks whose positions lie between those characteristic of pure wurtzite CdSe and those of pure wurtzite CdS and ZnS, which is expected for the CdSe/CdS/ZnS core/shell/shell system. In order to test the quality of the shell, an aromatic thiol (4-methylbenzenethiol), known to act as an efficient fluorescence quencher, was added to the core and core/shell/shell nanocrystals. For the CdSe core nanocrystals, no PL signal is measurable after addition of the quencher, while the core/shell/shell sample retains more than 80% of its initial PL intensity (Figure 6).

## Conclusions

The DOE method is an appropriate tool for the optimization of the synthesis of semiconductor nanocrystals. Nevertheless, the success of the method relies on an appropriate choice of the experimental parameters to be studied and definition of their levels. On the other hand, the definition of a suitable experimental plan is largely facilitated by the large body of literature existing on the synthesis of various types of nanocrystals. Using the DOE method, within a relatively low number of experiments, the influence of the different experimental parameters and their interactions can be studied. This knowledge enables the rational design of the synthesis of nanocrystals having a desired mean size and low size distribution, while maximizing at the same time the reaction yield. Therefore the DOE approach is of particular interest when the scale-up of the synthesis is aimed. In the case of CdSe nanocrystals, the synthesis can be scaled up by a factor of 20, while the obtained samples essentially maintain the characteristics of those obtained with the small-scale synthesis. In particular, the size distribution is kept below 10% when using a batch reactor, mechanical stirring, and rapid precursor injection via a peristaltic pump. We do not see any physical limit comprising the scale-up of the same type of synthesis to even larger quantities (kilogram scale). Equally, the growth of a CdS/ZnS double shell on CdSe nanocrystals follows exactly the same trend when carried out on the gram scale as in the case of syntheses with a yield below 100 mg. In recent years, a large number of different types of nanocrystals have been synthesized using the so-called "heating-up method" where all precursors are mixed at ambient temperature and heated subsequently [38-40]. In this case, the scale-up is even simpler than in the case of the hot-injection method, as the delicate and sometimes hazardous fast injection is no longer necessary.

## Methods

All reagents were purchased in the highest purity available from Sigma-Aldrich Chimie S.a.r.l. (Lyon, France), except oleylamine (Fisher Scientific, Illkirch, France), and used without further purification. Zinc and cadmium etylxanthate (Zn(EX)_2_, Cd(EX)_2_) were synthesized according to a reported procedure [26]. The reaction yield was calculated with respect to the conversion of the minority precursor (Cd or Se), after multiplying the concentration of the nanocrystals dispersed in hexanes with the number of CdSe monomer units per nanocrystal. The concentration of the nanocrystals was obtained by using the correlations between diameter, size, and molar exctinction coefficient listed in [41] and using the data from [12].

### General synthesis method of CdSe nanocrystals

In a 50-mL three-neck flask equipped with a condenser, a mixture of CdO, stabilizing ligands (oleic acid (OA) or stearic acid (SA)), and the solvent (ODE or a mixture of ODE and oleylamine) was degassed in primary vacuum for 30 min and then heated under argon to the reaction temperature (250 or 300°C). The mixture was left at this temperature till it became colorless, which generally took a few minutes. A 0.4 M solution of selenium powder in TOP or TBP, diluted with ODE or with ODE/oleylamine (so that the injected volume represents half of the volume of the Cd precursor solution) was then swiftly injected into the reaction flask. Aliquots were taken at different time intervals, diluted with chloroform and characterized by UV-visible and PL spectroscopy. The reaction was stopped after 18 min by removing the heating source (molten salt or graphite flake bath). The mean size and size distribution of the obtained nanocrystals were determined using TEM.

#### Synthesis of 3.6-nm CdSe nanocrystals

CdO (0.4 mmol), 10 mmol of stearic acid, 3.3 mL (10 mmol) of oleylamine, and 20 mL of ODE were mixed in a three-neck flask equipped with a condenser and degassed for 15 min. Then the flask was refilled with argon and heated to 250°C. Then 5 mL of a 0.4 M TOP-Se solution, diluted with 8.3 mL of ODE, was swiftly injected into the hot solution. For the purification, a mixture of 2.5 mL of methanol and 2.5 mL of chloroform is added to the reaction mixture, followed by the addition of 50 mL of acetone. In order to avoid the solidification/precipitation of excess stearic acid, the purification should be carried out when the reaction mixture has reached a temperature of around 80°C after removing the heating source. The nanocrystals are isolated by centrifugation at 4,000 rpm for 10 min and redispersed in hexanes before repeating the described cleaning cycle a second time.

UV-vis, 562 nm; PL, 584 nm (FWHM, 28.3 nm); yield, 95%; QY, 7%.

#### Synthesis of the 4.5-nm CdSe nanocrystals

Synthesis and purification were carried out in the same way as for the 3.7-nm nanocrystals except that the quantities were 0.4 mmol of CdO, 10 mmol of stearic acid, 14 mL (42.5 mmol) of oleylamine, and 9 mL of ODE. The injection mixture was composed of 5 mL of a 0.4 M TOP-Se solution, diluted with 3.3 mL of ODE and 5 mL of oleylamine.

UV-vis, 594 nm; PL, 614 nm (FWHM, 27.5 nm); yield, 80%; QY, 30%.

#### Synthesis of the 6.1-nm CdSe nanocrystals

Synthesis and purification were carried out in the same way as for the 3.7-nm nanocrystals except that the quantities were 0.8 mmol of CdO, 40 mmol of stearic acid, 5.3 mL of ODE and the injection mixture was composed of 1 mL of a 0.4 M TOP-Se solution, diluted with 8.5 mL of ODE. Note that in this case, no oleylamine is added, resulting in comparably low fluorescence QY. The temperature for this synthesis was 300°C.

UV-vis, 630 nm; PL, 643 nm (FWHM, 30 nm); yield, 60%; QY, 2%.

### Gram-scale syntheses

#### Synthesis of 5-nm CdSe nanocrystals

Before the synthesis, the whole setup is degassed for 1 h with a primary vacuum pump. Then, 8 mmol of CdSt_2_, 184 mmol of SA, 280 mL (0.85 mol) of oleylamine, and 186 mL of ODE are introduced in the 2-L reactor and degassed for 45 min. The reactor is then refilled with argon and heated to 250°C. At this temperature, 100 mL of a 0.4 M TOP-Se solution are swiftly (within 1 s) injected using a peristaltic pump. The mixture is maintained at 250°C for 15 min and then the heating source (molten salt or graphite flakes bath) is removed.

Nanocrystals are purified by filtration with a glass filter column (G3) before the mixture reaches ambient temperature in order to avoid the solidification/precipitation of excess stearic acid. Acetone (500 mL) is slowly added when the temperature reaches 80°C, followed by 200 mL of ethanol at 50°C and further 300 mL of acetone. The nanocrystals are redispersed in hexanes in order to separate leading to decantation of eventually remaining free stearic acid within some days.

UV-vis, 600 nm; PL, 614 nm (FWHM, 31.7 nm); yield, 2.3 g; (93%); QY, 37.6%.

#### Synthesis of 4-nm CdSe nanocrystals

Synthesis and purification were carried out in the same way as for the 5-nm nanocrystals, except that the quantities are 8 mmol of CdSt_2_, 184 mmol of SA, 65.8 mL (200 mmol) of oleylamine, 400 mL of ODE, and 100 mL of the 0.4 M TOP-Se solution.

UV-vis, 586 nm; PL, 597 nm (FWHM, 31.5 nm); yield, 2.1 g (88%); QY, 8.9%.

#### Synthesis of 6.7-nm CdSe nanocrystals

Synthesis and purification were carried out in the same way as for the 5-nm nanocrystals, except that the quantities are 16 mmol of CdSt_2_, 784 mmol of SA and 107 mL of ODE. The injected mixture contains 170 mL of ODE and 20 mL of TOP-Se 0.4 M. The injection temperature is 290°C and growth temperature 280°C.

UV-vis, 636 nm; PL, 648 nm (FWHM, 32 nm); yield, 2 g (62%); QY, 1.8%.

### CdS/ZnS shell growth on the 4-nm CdSe nanocrystals

NC (5 μmol) in 5 ml hexanes are put in the 2-L reactor together with 285 mL of ODE and 275 mL of oleylamine, and degassed under primary vacuum for 30 min. The reactor is then refilled with argon and heated to 200°C. At this temperature, 0.62 mmol of Cd(EX)_2 _in 7.8 mL of TOP and 1.86 mmol of CdSt_2 _in 21.7 mL of ODE (corresponding to the estimated quantity for 1.3 CdS monolayers) are injected for 30 min using a syringe-pump. The temperature is then set to 235°C for 10 min, followed by the addition of 2.5 mmol of Zn(EX)_2 _in 30 mL of TOP and 7.5 mmol ZnSt_2 _in 100 mL of ODE (corresponding to the estimated quantity for 2.5 ZnS monolayers) within 45 min.

UV-vis, 582 nm; PL, 592 nm (FWHM, 37.9 nm); QY, 81%; quantity, 1.28 g.

### Characterization techniques

Solution UV-vis spectra were recorded on a HP 8452A spectrometer (wavelength range 190-820 nm). Photoluminescence measurements were carried out using a Hitachi F-4500 spectrofluorometer (Hitachi Co., Tokyo, Japan) (Figures 3 and 4) or an AvaSpec-2048-2 spectrofluorometer (Avantes BV, Eerbeek, The Netherlands) and a blue LED (400 nm) for excitation (Figures 1 and 6). TEM images were obtained with a JEOL 4000EX microscope (JEOL, Tokyo, Japan). Powder X-ray diffraction was performed with a Philips X'Pert MPD diffractometer (Philips, Amsterdam, The Netherlands) using a Co X-ray source operated at 50 kV and 35 mA with a secondary graphite monochromator.

### Measurement of the fluorescence quantum yield

The absolute fluorescence QY of the nanocrystals was determined by comparison with a freshly prepared solution of Rhodamine 6G in ethanol (QY 95%), using the following formula:

with *Φ *being the QY, grad the gradient (slope) of the plot of the integrated fluorescence intensity *vs*. absorbance, and *n *the refractive index of the solvent (1.375 for hexanes, 1.36 for ethanol).

Purified samples of CdSe or CdSe/CdS/ZnS nanocrystals in hexanes were put into a 1-cm quartz cuvette and diluted until the absorbance at the first excitonic was below 0.1. At least three samples of different concentration were prepared. The absorbance of the standard was adjusted to be equal to each nanocrystals' dispersion at the PL excitation wavelength.

## Competing interests

The authors declare that they have no competing interests.

## Authors' contributions

MP and PR conceived of the experimental plan and MP carried out the syntheses of the core CdSe nanocrystals. NN performed the large-scale syntheses and characterization of the CdSe/CdS/ZnS core/shell/shell samples. OR participated in the design of the study. PR supervised the synthesis and carried out the TEM characterization. MP and PR prepared the manuscript. All authors read and approved the final manuscript.
